# High level of susceptibility to human TRIM5α conferred by HIV-2 capsid sequences

**DOI:** 10.1186/1742-4690-10-50

**Published:** 2013-05-06

**Authors:** Junko S Takeuchi, Benjamin Perche, Julie Migraine, Séverine Mercier-Delarue, Diane Ponscarme, François Simon, François Clavel, Béatrice Labrosse

**Affiliations:** 1 INSERM U941, Paris 75010, France; 2 Institut Universitaire d’Hématologie, Université Paris Diderot, Hôpital Saint-Louis, Paris 75010, France; 3 Laboratoire de Microbiologie, Hôpital Saint-Louis, Paris 75010, France; 4 Service des Maladies Infectieuses et Tropicales, Assistance Publique—Hôpitaux de Paris, Hôpital Saint-Louis, Paris 75010, France

**Keywords:** Primary HIV-2 strains, Human TRIM5α restriction factor, Capsid sequences

## Abstract

**Background:**

HIV-2, which was transmitted to humans from a distant primate species (sooty mangabey), differs remarkably from HIV-1 in its infectivity, transmissibility and pathogenicity. We have tested the possibility that a greater susceptibility of HIV-2 capsid (CA) to the human restriction factor TRIM5α (hTRIM5α) could contribute to these differences.

**Results:**

We constructed recombinant clones expressing CA from a variety of HIV-2 viruses in the context of HIV-1 NL4-3-luciferase. CA sequences were amplified from the plasma of HIV-2 infected patients, including 8 subtype A and 7 subtype B viruses. CA from 6 non-epidemic HIV-2 subtypes, 3 HIV-2 CRF01_AB recombinants and 4 SIVsmm viruses were also tested. Susceptibility to hTRIM5α was measured by comparing single-cycle infectivity in human target cells expressing hTRIM5α to that measured in cells in which hTRIM5α activity was inhibited by overexpression of hTRIM5γ.

The insertion of HIV-2 CA sequences in the context of HIV-1 did not affect expression and maturation of the HIV-2 CA protein. The level of susceptibility hTRIM5α expressed by viruses carrying HIV-2 CA sequences was up to 9-fold higher than that of HIV-1 NL4-3 and markedly higher than a panel of primary HIV-1 CA sequences. This phenotype was found both for viruses carrying CA from primary HIV-2 sequences and viruses carrying CA from laboratory-adapted HIV-2 clones. High hTRIM5α susceptibility was found in all HIV-2 subtypes. In this series of viruses, susceptibility to hTRIM5α was not significantly affected by the presence of a proline at position 119 or by the number of prolines at positions 119, 159 or 178 in HIV-2 CA. No significant correlation was found between HIV-2 viremia and sensitivity to hTRIM5α.

**Conclusions:**

HIV-2 capsid sequences expressed high levels of susceptibility to hTRIM5α. This property, common to all HIV-2 sequences tested, may contribute in part to the lower replication and pathogenicity of this virus in humans.

## Background

Primate species have evolved under unrelenting selective pressure by pathogenic lentiviruses. In response to lentiviral infections, primates have selected a number of restriction factors whose antiviral activity has subsequently become both species- and virus-specific. TRIM5α, a restriction factor expressed by all primate species, exerts its antiviral activity following entry through recognition of the incoming lentiviral capsid, which leads to both premature destabilization of the capsid structure and abortive reverse transcription, as well as induction of a cascade of signal transduction events that is believed to promote antiviral innate immune responses [1-5]. Human TRIM5α (hTRIM5α), which appears to have been selected under pressure by one or more ancient and presumably extinct virus(es), only exerts limited restriction activity on the lentiviruses that currently infect primate species [2,6,7]. In particular, the antiviral activity of hTRIM5α on HIV-1 is relatively low [8-12], with a few exceptions such as some of the viral variants that have developed CTL resistance mutations in CA epitopes presented by HLA-B*57 or -B*27 [13]. Overall, the natural expression of TRIM5α in human cells did not protect our species from pandemic-scale infection by HIV-1, following initial transmission of the virus to humans from SIVcpz-infected chimpanzees.

The chimpanzee species from which HIV-1 originates (*Pan troglodytes troglodytes*) is relatively closely related to humans [14], suggesting that little adaptation may have been needed on the part of HIV-1 to resist the antiviral activity of hTRIM5α and thereby propagate on an epidemic scale in humans [15,16]. In contrast, HIV-2, the other human lentivirus, is the result of transmission of SIVsmm, a virus that naturally infects sooty mangabeys (*Cercopithecus torquatus atys*) in Western Africa [17-19]. The profile of HIV-2 infection in humans is quite different from that observed with HIV-1. First, the level of viral production, as measured by HIV-2 RNA in plasma, is considerably lower than that of HIV-1 [20,21], and also remarkably low compared to that of SIVsmm in its natural host [22-24]. Second, the course of HIV-2 infection in humans is clearly longer and less pathogenic than that of HIV-1, with a protracted asymptomatic period followed, in some cases, by deterioration of CD4^+^ T lymphocyte counts and immune deficiency [25-27]. Third, HIV-2 transmission is less efficient than HIV-1 [28,29]. The geographic range of the HIV-2 epidemic, essentially restricted to West Africa, has not significantly changed since HIV-2 was first identified, while the range of HIV-1 infection has rapidly widened over this time period, yielding the current pandemic stage. In several West African countries where HIV-2 was predominant in the late 1980s, HIV-1 has now become the dominant human lentiviral infection [30,31]. Another strong indicator of the lower transmissibility of HIV-2 is the markedly lower rate of mother-to-child transmission in absence of preventive therapy, compared to that of HIV-1 [32,33].

Several mechanisms have been invoked to explain these differences: i) an intrinsic lower infectivity and pathogenicity of HIV-2, an hypothesis that is not consistent with the high level of SIVsmm replication in sooty mangabeys; ii) a more efficient control of HIV-2 replication in humans owing to a better induction or higher efficacy of human adaptive or innate immune responses; iii) a higher sensitivity of HIV-2 to human restriction factors, possibly due to the fact that HIV-2 has been transmitted to humans from a more distantly related simian species expressing divergent restrictions factors. The latter hypothesis would be consistent with the idea that HIV-2 has had a much longer time to adapt to mangabey restriction factors than to human restriction factors, if indeed the transmission of this virus to our species is a relatively recent event. In this regard, HIV-2 is divided into more than 8 subtypes [34-37], which, unlike HIV-1 subtypes, but similar to the HIV-1 M, N, O and P groups, generally correspond to separate transmission events from non-human primate to man. Subtypes A and B, which may have originated from the same transmission event, are the dominant, so-called “epidemic” subtypes, and are responsible for the vast majority of HIV-2 infections. Other subtypes are mostly sporadic, as they are represented by only a few or even a single viral isolate.

Susceptibility of HIV-2 to human restriction factors has not been extensively evaluated, and viral isolates that had been adapted for replication in cultures of primary or transformed human cells have often been used. In particular, analyses on HIV-2 susceptibility to hTRIM5α have been essentially restricted to studies on HIV-2 ROD [38,39], a highy laboratory-adapted HIV-2 isolate [40], although a limited number of primary isolates have also been studied using non-quantitative assays [41]. In spite of this lack of direct information on the susceptibility of primary HIV-2 strains to hTRIM5α, recent correlative studies have suggested that inter-strain differences in susceptibility to hTRIM5α or other CA-targeting restriction factors, as reflected by specific polymorphisms in the HIV-2 CA sequence, could contribute to differences in the clinical progression of HIV-2 infection [42]. In this study, we have examined the antiviral activity of hTRIM5α on recombinant viruses expressing a wide panel of HIV-2 CA proteins directly amplified from the plasma of infected patients. We conclude that HIV-2 capsids have higher susceptibility to hTRIM5α than what is generally observed with HIV-1. The level of susceptibility of HIV-2 CA to hTRIM5α is comparable between different strains and different subtypes of this virus, and is also comparable to that of the SIVsmm CA. Thus, although susceptibility of HIV-2 to hTRIM5α does not appear to play a determinant role in the differences in pathogenic profiles observed among HIV-2 infected patients, it may contribute to the overall lower rate of replication and propagation of this virus in humans.

## Results

### Maturation and biological properties of HIV-1 NL4-3-derived chimeric viruses expressing HIV-2 CA sequences

**Figure 1:**
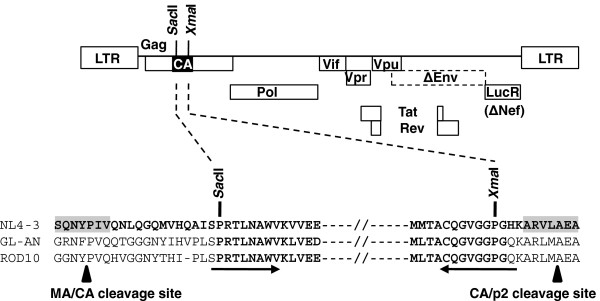
**Chimeric viruses carrying HIV-2 CA sequences.** The HIV-1 pNL4-3-ΔENV-LucR vector, in which the *Sac*II and *Xma*I restriction sites were introduced, was used as background to express a variety of HIV-2 and SIV CA sequences. The aminoacid alignment of the extended capsid-coding region of the HIV-1 NL4-3, and the HIV-2 strains GL-AN and ROD10 is shown. The HIV-1 Gag sequences recognized by the HIV-1 protease are highlighted in grey, and the MA/CA and CA/p2 cleavage sites are indicated. The arrows underline the sequences of primers carrying the *Sac*II and *Xma*I restriction sites, which were used to clone the HIV-2 CA sequences. The aminoacids present into wild-type or chimeric pNL4-3-ΔENV-LucR are in bold. MA, *matrix*; CA, *capsid*.

To examine the susceptibility of primary HIV-2 to hTRIM5α, we constructed chimeric clones expressing plasma-derived HIV-2 CA coding sequences in the context of an HIV-1 backbone. The backbone was pNL4-3-ΔENV-LucR [43], which permits optimal virus production and highly sensitive quantification of HIV-1 infectivity on a single-cycle basis. As shown on Figure 1, HIV-2 or SIV CA coding sequences (aa 17 to 224) were inserted between the HIV-1 MA/CA and CA/p2 cleavage site sequences, thereby preserving the HIV-1 identity of these sequences, and allowing optimal proteolytic cleavage of the HIV-2 CA by the HIV-1 protease expressed by the pNL4-3-derived vector. The HIV-2 and SIV CA sequences were categorized as follows: i) CA from laboratory adapted HIV-2 strains GL-AN and ROD10; ii) CA from primary plasma-derived HIV-2 sequences, including 8 subtype A and 7 subtype B viruses, iii) CA from HIV-2 CRF01_ AB NMC (Nagoya Medical Center) 307, NMC716 and NMC842 viruses, iv) synthetic CA sequences from 6 non-epidemic HIV-2 (subtypes C to H), 4 SIVsmm strains, and SIVmac239, and v) CA from 9 primary HIV-1 belonging to subtypes B and CRF02_AG.

**Figure 2:**
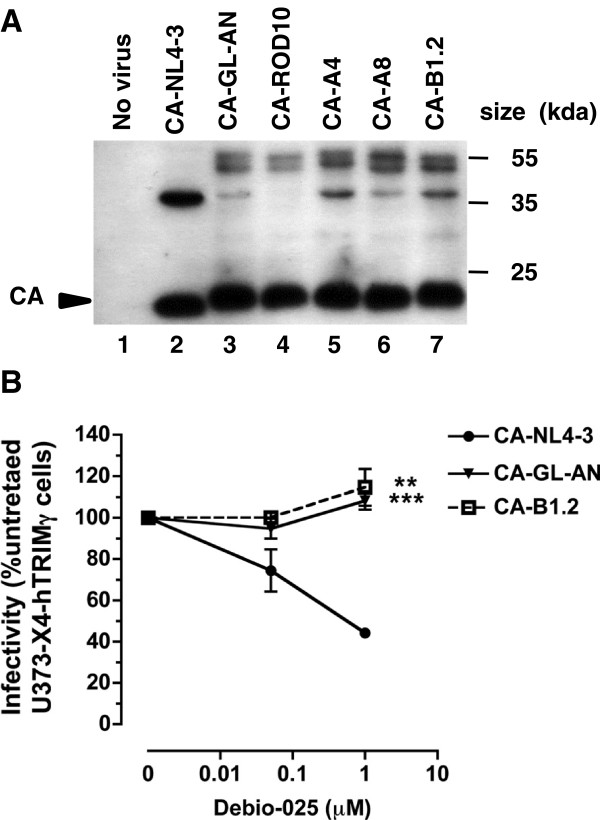
**CA proteins expression and sensitivity of chimeric viruses to Debio-025.** (**A**) Western blotting. The particle-associated proteins of HIV-1 NL4-3 (lane 2), HIV-1 expressing the capsid proteins from the two laboratory-adapted HIV-2 GL-AN (lane 2) and ROD10 (lane 3), from two subtype **A** clinical isolates HIV-2 CA-A4 (lane 5), A8 (lane 6), and from a subtype **B** clinical isolate HIV-2 B1.2 (lane 7) were analyzed. The viral supernatants of 293T transfected cells were concentrated by ultracentrifugation, through 20% sucrose cushion, resuspended and normalized by RT ELISA (0.3 ng) before western blot analysis using a mixture of serum derived from HIV-2 subtype A or B-infected patients. Lane 1: supernatant of untransfected 293T cells. The location of the CA proteins is shown on the left. The molecular mass marker (in kilodaltons) is indicated on the right. Results are representative of three independent experiments. (**B**) Effect of Debio-025 on viral infectivity. U373-X4 cells overexpressing hTRIM5γ were pretreated for 20 h with 100 U/ml IFN-α, and infected with equal amounts (0.2 ng RT) of the indicated VSV-G-pseudotyped chimeric viruses in the presence of the indicated concentrations of Debio-025. Forty hours after infection, medium was removed, the cells were lysed, and luciferase activity was determined by luminometry. The results, which are expressed relative to the infectivity of that of untreated cultures, are the mean ± SEM for 3 experiments. Asterisks indicate the results of statistical analyses comparing infectivity of chimeric virus to that of NL4-3, as determined using the non-parametric Mann–Whitney test: **, *p* = 0.0014; ***, *p* = 0.0001.

CA maturation in the chimeric viruses was analyzed by western blot after purification by ultracentrifugation of virions over a 20% sucrose cushion, normalization for RT content by ELISA and reaction with a mixture of human sera from HIV-2 positive patients. As shown on Figure 2A, chimeric virions contained high amounts of fully mature p26 CA protein, together with small amounts of higher molecular weight Gag cleavage intermediates. The ratio of fully processed CA to cleavage intermediates was comparable to that observed for pNL4-3 particles, although the pattern of incompletely processed Gag proteins was different in the two virion preparations. The profile of incompletely cleaved Gag proteins was similar among the different HIV-2 CA-expressing chimeric virions studied.

To further validate that the mature capsids of these chimeric viruses had biological properties expected from HIV-2, we also examined their susceptibility to the cyclophilin A (CypA) antagonist Debio-025. Indeed, while most HIV-1 strains have been described as being susceptible to CypA antagonists, the replication of HIV-2 has been consistently reported to be unaffected by these antagonists, a property that is related to specific genetic traits of its CA protein [44-46]. As expected, and in contrast with the HIV-1 control virus, no detectable inhibition of viral infectivity by Debio-025 was observed for the two chimeric viruses carrying HIV-2 CA sequences tested (Figure 2B). This observation indicates that these viruses had acquired biological properties consistent with that of expression of a functional HIV-2 CA protein, making them suitable for hTRIM5α susceptibility testing.

### Infectivity and susceptibility to hTRIM5α of viruses expressing HIV-2 CA

**Figure 3:**
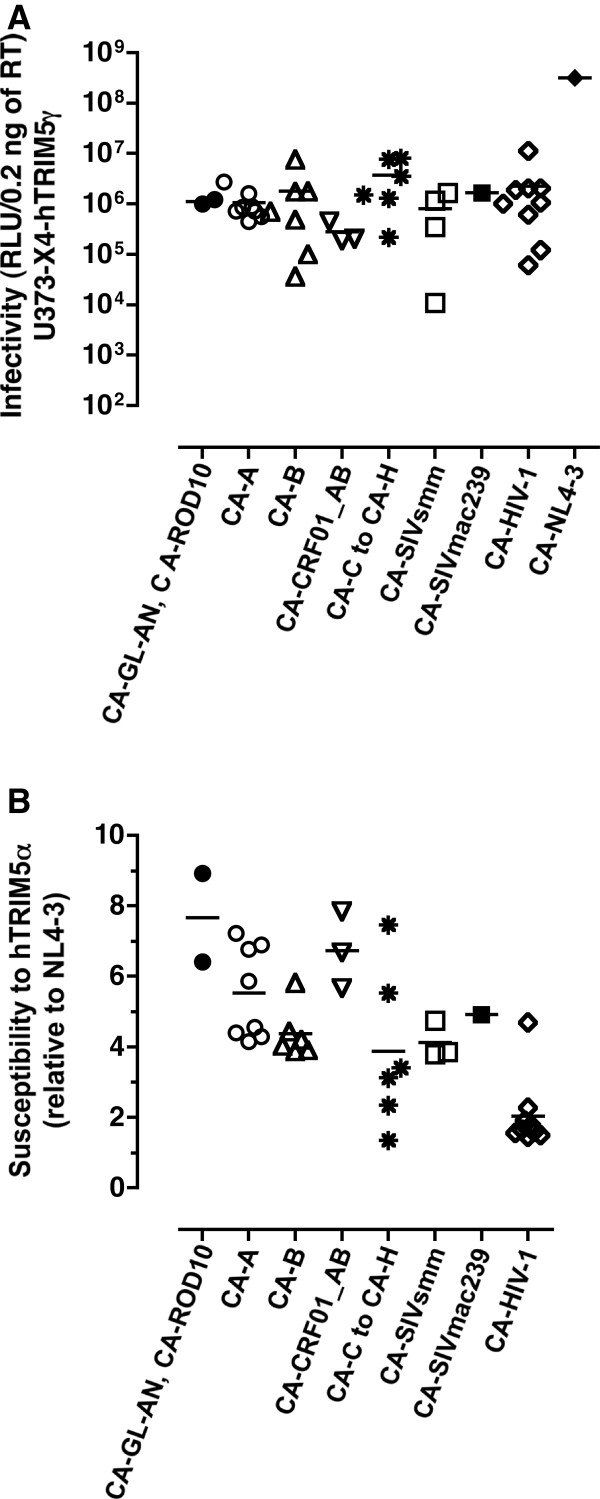
**Measurement of viral infectivity and susceptibility to hTRIM5α.** The U373-X4-hTRIM5γ and U373-X4-LacZ target cells were pretreated for 20 h with 100 U/ml IFN-α before their infection with 0.2 ng RT of chimeric viruses. The viruses were grouped according to the origin of their CA proteins: laboratory-adapted HIV-2 strains (n=2), HIV-2 clinical isolates from subtype **A** (n=8) or from subtype **B** (n=7), CRF01_AB (n=3), synthetic sequences from HIV-2 viruses from non-epidemic subtypes C to H (n=6), synthetic sequences from SIVsmm (n=4) and from SIVmac239, primary HIV-1 CA sequences from patient plasma (n=9). Forty hours after luciferase activity was determined by luminometry. (**A**) Infectivity values of chimeric viruses measured in the U373-X4 target cells in which hTRIM5α activity had been inhibited by overexpression of hTRIM5γ, which are expressed as RLU/0.2 ng of RT. (**B**) Susceptibility values of chimeric viruses to hTRIM5α were expressed as a ratio [(RLU for hTRIM5γ cells)/(RLU for LacZ cells)], and normalized to that obtained for NL4-3. The results shown are the mean ± SEM for 3 independent experiments.

Infectivity of recombinant viruses carrying HIV-2 and SIV CA sequences was tested in a luciferase-based single-cycle assay in U373-X4-hTRIM5γ cells, in which the antiviral activity of hTRIM5α is offset by stable overexpression of hTRIM5γ. Stocks of viruses were produced by transfection of 293T cells and normalized for RT content by ELISA. To ensure that no viral propagation would occur in the target cell cultures, virus particles were pseudotyped with VSV-G. The results of viral infectivity obtained using U373-X4-hTRIM5α cells, which were pretreated with IFNα before infection, are presented on Figure 3A. The viruses were grouped according to the origin of their CA proteins: laboratory-adapted HIV-2 strains (n=2), HIV-2 clinical isolates from subtype A (n=8) or from subtype B (n=7), CRF01_AB (n=3), synthetic sequences from HIV-2 viruses from non-epidemic subtypes C to H (n=6), synthetic sequences from SIVsmm (n=4) and from SIVmac239. The results were compared to those obtained for NL4-3 and to those from a panel of recombinant viruses expressing CA from a panel of primary HIV-1 plasma sequences. This panel included recombinant virus NRC10-5, a previously described NL4-3 derived virus carrying the CA sequences from a clinical isolate that expresses high susceptibility to hTRIM5α [12,13]. For the HIV-2 CA recombinants, infectivity values ranged from 1×10^4^ to 8×10^6^ relative light units (RLU)/0.2 ng of RT (Figure 3A), but no virus group displayed infectivity levels that differed significantly from those of any of the other groups (*p* > 0.05). The mean infectivity values of viruses carrying primary HIV-1 CA and NL4-3 CA were 6×10^4^ and 3×10^8^ RLU/0.2 ng of RT, respectively. Among the panel of our chimeric viruses, one of the seven viruses carrying CA from a primary HIV-2 subtype B and one of the four viruses carrying CA from a SIVsmm strain could not be tested phenotypically for their susceptibility to hTRIM5α since their infectivities were less than 4×10^4^ RLU/0.2 ng of RT (Figure 3A).

Susceptibility to hTRIM5α was expressed as the ratio of viral infectivity measured in U373-X4-hTRIM5γ cells to that measured in U373-X4-LacZ control cells. This method has been shown by our laboratory to be fully valid for hTRIM5α susceptibility testing of viruses carrying primary HIV-1 Gag sequences [12,13]. Susceptibility to hTRIM5α of viruses expressing HIV-2 CA is shown on Figure 3B. Overall, susceptibility of most HIV-2 CA-carrying viruses was greater than that measured for HIV-1 NL4-3. Highest susceptibility values were found with viruses expressing CA from laboratory-adapted HIV-2 strains GL-AN and ROD10, respectively 6.4-fold (*p* = 0.0086) and 8.9-fold (*p* = 0.0016) more susceptible to hTRIM5α than NL4-3. The lowest susceptibility values were measured in viruses CA from non-epidemic HIV-2 subtypes (mean = 3.3). Susceptibility values for this series of viruses were markedly heterogeneous, ranging from 7.5-fold for CA from HIV-2 subtype F to 1.4-fold with CA from HIV-2 subtype G. Such heterogeneity was not observed for viruses expressing SIVsmm CA. As expected, hTRIM5α susceptibility values obtained for viruses carrying primary HIV-1 CA was low (mean = 2.0-fold), including 8 viruses for which susceptibility values ranged 1.4- to 2.3-fold. Consistent with previous observations, the susceptibility of NRC-10, which expresses mutations conferring CTL resistance in the HLA-B*27-targeted epitope KK10, was 4.7-fold. Overall, susceptibility to hTRIM5α of primary HIV-1 CA-carrying viruses was significantly lower than that measured for recombinant viruses carrying CA from either HIV-2 subtype A (*p* = 0.001), HIV-2 subtype B (*p* = 0.008) or HIV-2 CRF01_ AB (*p* = 0.009), respectively.

### Absence of correlation between plasma viral loads and hTRIM5α susceptibility

**Figure 4:**
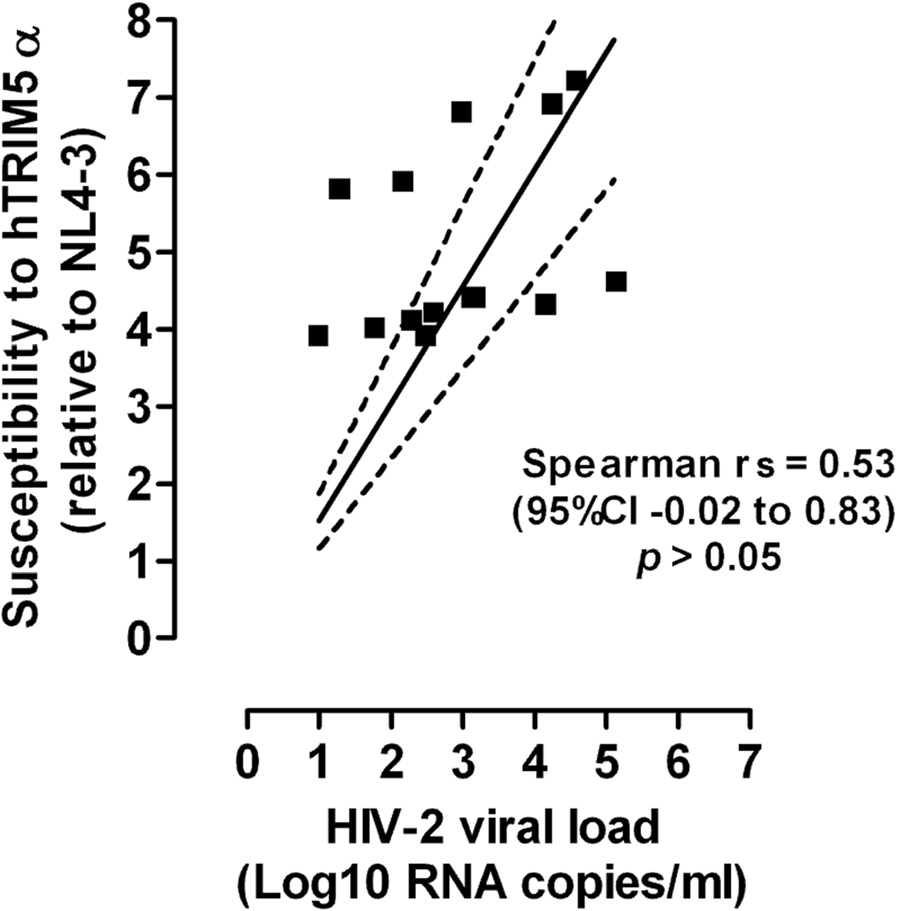
**Plasma viral loads and hTRIM5α susceptibility.** Quantification of plasma HIV-2 viral load (Log_10_ RNA copies/ml) was performed on plasma samples obtained from 8 HIV-2 subtype A-infected and 6 HIV-2 subtype B-infected patients for whom we measured their sensitivity to hTRIM5α. Linear regression curve (continuous line) and 95% CI (dotted line) are presented. The correlation coefficient and *p* value were calculated by Spearman test.

Quantification of plasma HIV-2 viral load (VL) was performed by a method derived from that described by Damond et al. [34], using a modified set of primers targeting the 5'-long terminal repeat (5'-LTR) region with a detection limit of 10 HIV-2 RNA copies/ml (Figure 4). The quantification of viral load was performed on plasma samples obtained from 8 HIV-2 subtype A-infected and 6 HIV-2 subtype B-infected patients for whom we were able to reconstruct chimeric viruses and measure their sensitivity to hTRIM5α. HIV-2 viral loads ranged from 1 to 5.15 Log_10_ RNA copies/ml, and were higher for HIV-2 subtype A-infected patients than for HIV-2 subtype B-infected patients (mean values of 3.6 Log_10_ and 2.1 Log10 RNA copies/ml, respectively, *p* < 0.02). However, no correlation was found between HIV-2 viral load and susceptibility to hTRIM5α of the chimeric viruses carrying the CA from these HIV-2 infected patients.

### Impact of polymorphisms at positions 119, 159 and 178 of CA to hTRIM5α susceptibility

Song et *al.*[41] reported that HIV-2 propagation in human cells overexpressing hTRIM5α is less efficient for viruses carrying a proline at position 119 of CA protein, as compared to that of viruses expressing a glutamine or an alanine at this position. More recently, studies by Onyango et *al*., evaluating HIV-2-infected patients from Guinea Bissau, found that the concommittent presence of a proline at CA positions 119, 159 and 178 is more frequent in subjects with low VL while non-proline aminoacids at these three sites are more frequent in subjects with high VL [42]. The authors suggested that this relationship could be explained by differences in susceptibility to hTRIM5α.

**Figure 5:**
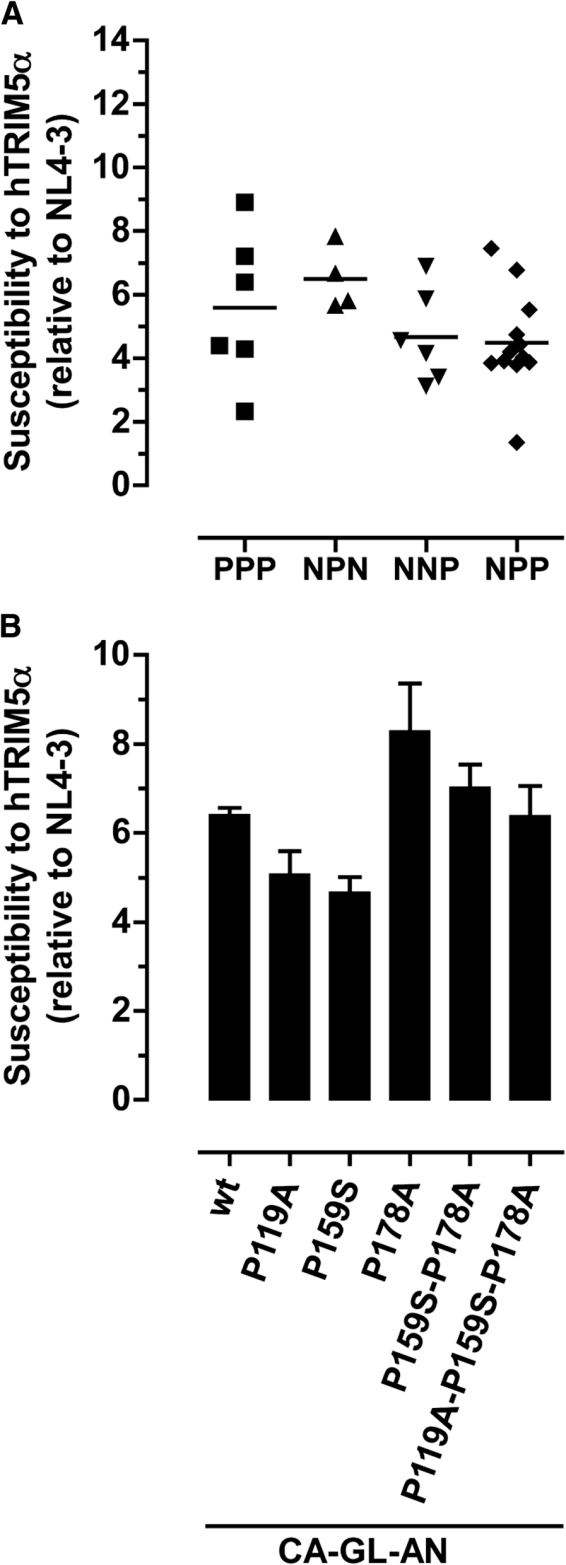
**Impact of the CA residues 119, 159, and 178 on hTRIM5α susceptibility.** (**A**) hTRIM5α susceptibility of HIV-1 chimeric viruses that are stratified by the presence of proline (P) or no proline residue (N) at each of the CA positions 119, 159 and 178. For example, PPP = proline residues at positions 119, 159 and 178, NNN = no proline at the three positions. (**B**) hTRIM5α susceptibility values of HIV-1 chimeric virus carrying GL-AN capsid sequence (CA-GL-AN) and of its corresponding mutants CA-GL-AN P119A, CA-GL-AN P159S, CA-GL-AN P178A, CA-GL-AN P159S-P178A, and CA-GL-AN P119A- P159S-P178A were compared. The results shown are the mean ± SEM for 3 independent experiments.

To explore the potential impact of these variations on the susceptibility to hTRIM5α, we grouped our HIV-1 chimeric viruses according to whether the residues at CA positions 119, 159 and 178 were a proline (P) or non-proline (N) amino acid. For these studies, the panel of HIV-1 chimeric viruses studied was increased by including of variants harboring CA sequences derived from minority populations present in the plasma from some of the infected patients. As shown on Figure 5A, the mean hTRIM5α susceptibility ranged from 4.4 (NPP viruses) to 7.8 (NPN viruses). Considerable overlap between the different groups was observed, however, and these differences were not statistically significant. In addition, no correlation was observed between the number of proline residues present at these three positions and hTRIM5α susceptibility.

We also analyzed the impact on hTRIM5α susceptibility of amino acid substitutions involving one or more of these proline residues. Mutagenesis was performed on the recombinant virus carrying CA from the GL-AN strain, which, like its parental strain GH123 [47], harbored proline residues at CA positions 119, 159 and 178. Modifying individually these proline residues (P119A, P159S, P178A) did not significantly change viral susceptibility to hTRIM5α, although the mutation P178A induced a 1.3-fold increase compared with that of CA-GL-AN (Figure 5B). Similarly, the double mutant P159S-P178A and the triple mutant P119A-P159S-P178A had comparable hTRIM5α susceptibilities (mean values of 7.0 and 6.4, respectively) that were not significantly different from that of the CA-GL-AN virus (mean value of 6.4).

## Discussion

The results presented here show that recombinant HIV-1 viruses carrying the CA protein from a panel of laboratory-adapted or primary HIV-2 strains express comparable levels of susceptibility to human hTRIM5α, which we found significantly increased compared to that of HIV-1. These recombinant viruses were found to produce normal amounts of fully proteolytically processed CA protein, the likely result of inserting the HIV-2 CA between cleavage site sequences that were spared in the cloning process and that are specific for cleavage by the HIV-1 protease expressed by the NL4-3 backbone. It is noteworthy that the resulting recombinant viruses were less infectious than the parental NL4-3 virus. This feature did not appear to be attributable to a CA proteolytic processing defect, and may be the consequence of some undefined structural effect. We provide strong evidence, however, that these viruses produced a capsid structure that is biologically relevant to that of full length HIV-2. Of note, no correlation was found between susceptibility to hTRIM5α and infectivity. Furthermore, the chimeric viruses displayed strong resistance to CypA antagonist Debio-025, a phenotype that is expected from viruses expressing a functional HIV-2 CA protein.

Unlike earlier studies on HIV-2 susceptibility to hTRIM5α, which mostly used partially quantitative propagative assays, our study was based a highly sensitive and reproducible single-cycle assay whose results have been shown to be fully consistent with those of earlier studies evaluating HIV-1. Human TRIM5α susceptibility was evaluated by quantifying single-cycle infectivity in U373 cells expressing IFN-enhanced levels of hTRIM5α and comparing it with that measured in cells in which the antiviral activity of hTRIM5α has been stably obliterated by overexpression of hTRIM5α, a truncated, inactive, trans-dominant isoform of hTRIM5α lacking a PRYSPRY domain. In previous work, it was established that this system was better suited for measuring hTRIM5α antiviral activity than partial disruption of hTRIM5α expression by a miRNA-based approach. To enhance their infectivity, virions were pseudotyped by the VSV-G envelope glycoprotein, which, although it directs virus entry through a route that differs from that of virions naturally expressing HIV-1 Env, does not affect their sensitivity to hTRIM5α. Using this system, we observed that almost all of the viruses expressing HIV-2 capsid from the panel tested here were significantly more susceptible to hTRIM5α than HIV-1. Little difference was found comparing viruses carrying CA from laboratory-adapted HIV-2 strains to viruses expressing CA from primary, plasma-derived HIV-2 sequences. Moreover, no significant difference was seen comparing viruses with HIV-2 CA derived from the two main epidemic subtypes (subtypes A and B), viruses with CA sequences from sporadic, non-epidemic subtypes or from a novel recombinant A/B CRF_01 subtype. Similar levels of susceptibility were also measured for viruses with CA from three different representatives of SIVsmm. Overall, these findings suggest that hTRIM5α did not constitute a strong barrier for transmission of SIVsmm to humans. If that were the case, one would have expected to see different levels of HIV-2 CA evolution and adaptation to hTRIM5α leading to differences in susceptibility according to the virus strain, with strongest adaptation in laboratory-adapted strains and lowest adaptation in sporadically transmitted non-A non-B subtypes and in SIVsmm.

Our study provides further evidence that susceptibility to hTRIM5α does not constitute a decisive factor for adaptation and replication of HIV-2 in humans. First, we failed to observe any significant correlation between susceptibility to hTRIM5α and HIV-2 plasma viral load, which was only measured in patients from the HIV-2 cohort infected with HIV-2 subtypes A or B. HIV-2 plasma RNA was measured using a quantitative PCR assay targeting well-conserved HIV-2 LTR sequences and yielding viral load levels that were clearly higher than those measured with the PCR assay targeting *gag* used in our prior studies. With this assay, most if not all tested patients displayed detectable viremia ranging from ≥ 10 to 141,254 copies/ml, with a median of 704 copies/ml. Second, our findings do not support the idea that the number and pattern of proline residues at positions 119, 159 and 178 of the HIV-2 CA can have a strong impact on sensitivity to hTRIM5α, and that certain of these patterns are associated with higher levels of HIV-2 replication and HIV-2 disease progression *in vivo*[42]. Among the panel of HIV-2 primary CA sequences tested in our study, no significant correlation appeared between the number and pattern of proline residues at these positions and either hTRIM5α sensitivity or viral load. Consistent with these findings, modifying the profile of these proline residues in a virus expressing CA from laboratory-adapted HIV-2 GL-AN did not have striking effects on its susceptibility to hTRIM5α. Thus, although changes in these residues may influence sensitivity to hTRIM5α in some isolates, this effect appears to be dependent on the CA context in which the modifications are introduced. Thus, the profile of proline residues cannot be used as a reliable surrogate marker for identifying viruses that are sensitive or resistant to hTRIM5α.

In conclusion, our findings reveal that hTRIM5α did not constitute a significant barrier to transmission of HIV-2 in humans and that no apparent adaptation of HIV-2 CA towards lower hTRIM5α susceptibility appears to have occurred either in vivo or during the in vitro propagation of HIV-2 in human cells. Most HIV-2 strains, however, display a significantly higher level of susceptibility to hTRIM5α compared to that of HIV-1. This lack of adaptation, in spite of higher susceptibility, may be explained by a high genetic barrier to adaptation in the context of the HIV-2/SIVsmm CA sequence. Such evolution may require the introduction of complex sets of genetic changes, and the sequential introduction of these mutations may be prevented if the virus encounters important troughs in intrinsic fitness and/or resistance to hTRIM5α during the acquisition of the necessary mutations. Thus, it remains possible that the higher susceptibility of HIV-2 to hTRIM5α participates to some extent to the lower pathogenicity and replication efficiency of this virus in humans. However, the lack of correlation between susceptibility to hTRIM5α and HIV-2 plasma viral load strongly suggests that other factors, independent from this restriction factor, may be altered during *in vivo* progression of HIV-2 disease.

## Conclusion

In conclusion, our studies indicate that the susceptibility to hTRIM5α of HIV-1 chimeric viruses carrying a wide panel of HIV-2 CA proteins was higher than what is generally observed with HIV-1. This level hTRIM5α susceptibility is comparable between different strains and different subtypes of HIV-2, and is also comparable to that of the SIVsmm CA. Furthermore, no significant correlation was found between HIV-2 viremia and sensitivity to hTRIM5α. Overall, these findings suggest that hTRIM5α did not constitute a decisive factor for adaptation and replication of HIV-2 in humans.

## Methods

### Reagents

Alpha interferon (IFNα) (Sigma-Aldrich) was dissolved in deionized water (final concentration, 2 × 10^5^ U/ml), and stored in aliquots at −80°C. Debio-025 (kindly provided by Debiopharma, Lausanne, Switzerland) was dissolved in dimethyl sulfoxide (DMSO) at a final concentration of 10 mM, and stored in aliquots at −80°C.

### Cell culture

293T and U373-X4 cell lines were cultured in Dulbecco’s modified Eagle’s medium supplemented with 10% fetal calf serum, 100 U/ml penicillin G and 100 μg/ml streptomycin. The two U373-X4 cell lines used as target cells, have previously been described [12]: (i) U373-X4 cells over-expressing human TRIM5γ (hTRIM5α cells), which has a dominant-negative effect against endogenous human TRIM5α; (ii) U373-X4 cells over-expressing *LacZ* (LacZ cells), which were used as a control. Human TRIM5γ cells and LacZ cells were maintained in the presence of 10 μg/ml puromycin, 100 μg/ml hygromcin B, and 8 μg/ml blasticidin.

### Construction of HIV-1 viruses expressing HIV-2 CA or SIV CA proteins

To generate recombinant viruses expressing CA sequences from HIV-2 and SIV viruses, we used the HIV-1 pNL4-3-ΔENV-LucR vector as background, which contains *Renilla* luciferase reporter gene in place of the HIV-1 *nef* locus and carries a large deletion in *env*[43]. To facilitate the construction of CA chimeric proviruses, *Sac*II and *Xma*I restriction sites were introduced at positions 49 and 670 of the CA domain of pNL4-3-ΔENV-LucR, respectively, using the QuickChange site-directed mutageneis kit (Stratagene), thereby creating pNL4-3-ΔENV-LucR-SX (Figure 1). The HIV-2 and SIV capsid sequences tested in this study were derived from different origins.

#### CA sequences from primary HIV-2 subtypes A and B

We used frozen plasma samples from 15 infected patients of the French ANRS HIV-2 cohort to generate recombinant viruses expressing CA sequences derived from either primary HIV-2 subtype A (CA-A1 to CA-A8) or subtype B (CA-B1 to CA-B7). To concentrate viral particles, plasma samples were ultracentrifugated (TLA 100.4 rotor; OptimaTM TLX ultracentrifuge; Beckman Instruments) at 164,000 × g for 30 min at 4°C in 3.2 ml polycarbonate tubes (Beckman), and RNA purification was performed using the QIAamp Viral RNA Mini kit (Qiagen). Extracted RNAs were directly used for reverse transcriptase-PCR (RT-PCR) amplification or stored at −80°C until use. The RT-PCR reaction was performed with the SuperScript III One-Step RT-PCR (Invitrogen) using the following primers: HIV-2-CA-F1 (5′-CCGCTGAGTCCCCGAACTCTAAAT) and HIV-2-CA-R1 (5′-CCTCCTTTAAGGCTTCTGCCATTA). For three RNA samples, the CA domain (CA-A2, CA-B3, and CA-B6) was amplified with the following primer set: HIV-2-CA-F2 (5′-GAAGTTGCGRGGCTTCTTTCCC) and HIV-2-CA-R2 (5′-GTAGACCAACAGCACCACCTAG). The nested PCR reaction was performed using the Accuprime Pfx Supermix (Invitrogen) and the primers: CA-*Sac*II-F (5′-AGCCCGCGGACCCTAAATGCCTGGGTAAA) and CA-*Xma*I-R (5′-TTTCTGCCCGGGGCCACCTACCCCCTG).

#### CA sequences from primary HIV-2 CRF01_AB

The plasmids coding the *gag* region of the HIV-2 CRF_01 AB originating from three different patients [48] [NMC (Nagoya Medical Center) 307, NMC716 and NMC842; kindly provided by Dr. Ibe were used to amplify the CA domain using the primers CA-*Sac*II-F and CA-*Xma*I-R.

#### CA sequences from laboratory-adapted HIV-2 ROD10 and GL-AN

The proviral molecular clones pGL-AN and pROD10 were kindly provided by Dr. Nguyen and Dr. Adachi [47,49] and by Dr. Peden [50], respectively. The corresponding CA sequences were amplified with the primers CA-*Sac*II-F and CA-*Xma*I-R. Five recombinant viruses carrying GL-AN capsid sequence with one or more mutations introduced by site-directed mutagenesis were constructed: P119A (CA-GL-AN P119A), P159S (CA-GL-AN P159S), P178A (CA-GL-AN P178A), P159S+P178A (CA-GL-AN P159S-P178A), and P119A+P159S+P178A (CA-GL-AN P119A-P159S-P178A).

Each PCR product was verified by agarose gel electrophoresis and column purified (Qiagen) prior to sequencing, and was subcloned into the pCR2*.*1 vector (TOPO TA cloning Kit, Invitrogen). Six to eight independent clones were sequenced for each bulk PCR product, and in most cases only clones possessing the sequence that matched the majority sequence were cloned into the HIV-1 pNL4-3-ΔENV-LucR-SX vector.

#### Synthetic CA sequences from non-epidemic HIV-2 and SIV

CA sequences derived from six non epidemic HIV-2 subtypes: subtype C (Genbank accession number L33077 [36]), D (L33083 [36]), E (L33087 [36]), F (U75441 [51]), G (AF208027 [52]), and H (AY530889 [35]), four SIVsmm (L09213 [53], AB553975 [54], U72748 [55], and X14307 [56]), and SIVmac239 (M33262 [57]), were commercially synthesized (GenScript). Since, the complete CA sequences of HIV-2 subtypes C, D, E, and F were not available from the Los Alamos database their C-terminal regions (~ 50 aminoacids) were complemented by a SIVsmm sequence (X14307 [56]).

### Quantification of plasma viral load

The HIV-2 viral load measurements were performed at Hôpital Saint-Louis using the real-time quantitative PCR method for measuring the HIV-2 RNA load as previously described [34], with the exception that a new set of primers overlapping the 5'-long terminal repeat (5'-LTR) region allowing to distinguish between HIV-2 subtypes A and B. Six of these patients were clinically considered as aviremic with viral loads below 200 RNA copies/ml.

### Production of chimeric viruses

The pNL4-3-ΔENV-LucR-SX vector or the corresponding chimeric proviruses (1 μg) and a VSV-G expression plasmid (phCMV-G, 0.1 μg) were cotransfected into 5×10^4^ 293T cells seeded into 6 well plates using jetPEI reagent (PolyPlus Transfection). Virus-containing culture supernatants were harvested 40 h after transfection, clarified by centrifugation to remove cell debris, assayed for their reverse transcriptase (RT) content using the HS-Lenti RT activity kit (Cavidi AB), and used directly for western blot analysis and infectivity assays.

### Western blotting

The VSV-G-pseudotyped HIV-1 NL4-3-derived particles were harvested 40 h post-transfection, purified and concentrated by centrifugation through a 20% sucrose cushion at 110,000 × g for 90 min using a TLA 100.4 rotor and an Optima TLX Ultracentrifuge (Beckman Coulter) and then resuspended in 50 μl Laemmli sample buffer (Bio-Rad). Particle-associated proteins corresponding to 0.3 ng of RT were separated by gel electrophoresis into a 12% SDS-PAGE gel, transferred to a PVDF membrane (Whatman), and blocked in Odyssey western blot blocker for 3 h at room temperature. To analyze the Gag processing pattern an to detect HIV-2 CA proteins, the membranes were probed with a mixture of sera derived from HIV-2 subtype A- and B-infected patients at a 1:2,000 dilution for 2 h at room temperature, washed, incubated with a HRP-conjugated goat anti-human IgG - H&L (ab6858, Abcam) at a 1:20,000 dilution for 1 h at room temperature, and detected using a ECL Advance western blotting detection Kit (RPN2135, GE healthcare).

### Measurement of viral infectivity

Each viral infectivity was measured by determining *Renilla* luciferase activity in target cells 40 h after infection, as previously described [12]. Briefly, the U373-X4-hTRIM5γ and/or the U373-X4-LacZ target cells (1 × 10^4^ cells) were initially plated in black-walled, clear bottom 96-well plates, cultured in the presence of 100 U/ml IFNα for 20 h to increase the expression of TRIM5α, and infected with 2 ng RT/ml of virus supernatant. After 40 hours, the supernatant of adherent cells was completely removed and 50 μl of 1X lysis buffer (Renilla Luciferase kit, Promega) were added to each well. Plates were maintained at room temperature for 30 min, after which wells were sequentially injected with 100 μl of luciferase substrate (Promega), and three seconds later, light emission (relative light units, RLU) was measured over a two sec interval using a luminometer (Varioskan Flash, Thermo scientific). Each sample was evaluated in triplicate and three independent experiments were performed. Results were expressed as mean for three experiments.

#### Effect of Debio-025 on viral infectivity

The U373-X4-hTRIM5γ target cells were pretreated for 20 h with 100 U/ml IFNα and with 0.05 μM or 1 μM Debio-025 for 15 min before viral infection (0.2 ng of RT). Infectivity values are expressed relative to those of cells not pretreated with Debio-025 and infected with the same virus.

#### Measurement of hTRIM5α susceptibility

The U373-X4-hTRIM5γ and the U373-X4-LacZ target cells were pretreated for 20 h with 100 U/ml IFNα before infection with 0.2 ng RT/ml of virus supernatant. The results were expressed as a ratio [(RLU for hTRIM5γ cells)/(RLU for LacZ cells)], and normalized to results obtained for NL4-3.

### Statistical analysis

All results are expressed as mean ± standard error of the mean (SEM). Comparisons between two groups were performed using the Mann–Whitney test; comparisons between multiple groups within a single series of experiments were performed using the Kruskal-Wallis test; post-test comparisons, performed only if *p* < 0.05, were performed using Dunn’s multiple comparison test. In all cases, GraphPad Prism 5 software was used.

## Competing interests

The authors declare that they have no competing interests.

## Authors’ contributions

SMD and FS designed and performed the plasma viral load quantification assay. DP clinically followed the HIV-2-infected patients and collected blood samples. JST, BP and JM performed experiments. JST, BL and FC participated in the design of the study, the analysis of the data and wrote the manuscript. All authors read and approved the final manuscript.

## Authors’ information

JST was supported by a fellowship from Sidaction.
